# In Vitro Effect of the Traditional Medicine Hainosan (Painongsan) on *Porphyromonas gingivalis*

**DOI:** 10.3390/medicines6020058

**Published:** 2019-05-20

**Authors:** Masaaki Minami, Hiroshi Takase, Masayo Taira, Toshiaki Makino

**Affiliations:** 1 Department of Bacteriology, Graduate School of Medical Sciences, Nagoya City University, 1 Kawasumi, Mizuho-ku, Nagoya 467-8601, Japan; 2 Core Laboratory, Graduate School of Medical Sciences, Nagoya City University, 1 Kawasumi, Mizuho-ku, Nagoya 467-8601, Japan; takase@med.nagoya-cu.ac.jp; 3 JPS Pharmaceutical Co. Ltd., 4-42-22 Higashiyamata, Tsuzuki-ku, Yokohama 224-0023, Japan; m-taira@jps-pharm.com; 4 Department of Pharmacognosy, Graduate School of Pharmaceutical Sciences, Nagoya City University, 3-1 Tanabe-Dori, Mizuho-ku, Nagoya 467-8603, Japan; makino@phar.nagoya-cu.ac.jp

**Keywords:** Hainosan, Platycodi Radix, platycodin D, *Porphyromonas gingivalis*, gingivitis, periodontitis

## Abstract

**Background**: Hainosan (Painongsan) is a traditional Japanese and Chinese medicine that is used to treat several purulent diseases, including gingivitis and periodontitis. This formulation contains three crude drug components: The dried immature fruit of *Citrus aurantium* (Aurantii Fructus Immaturus), the dried root of *Paeonia lactiflora* (Paeoniae Radix), and the dried root of *Platycodon grandiflorum* (Platycodi Radix). Here we evaluated the in vitro antibacterial effects of hainosan extract (HNS) and extracts of its three components against *Porphyromonas gingivalis*, one of the pathogenic bacteria that causes periodontitis. **Methods:** The antibacterial activities of HNS and its components were examined by counting the number of colony-forming units (CFUs) and through transmission electron microscopy. **Results:** We found that HNS had direct antibacterial activity against three *P. gingivalis* isolates (JCM12257, JCM8525, and JCM19600), with HNS-treated cells being significantly smaller than those of untreated bacteria. Extracts of Platycodi Radix and Paeoniae Radix significantly suppressed the growth of *P. gingivalis* in a dose-dependent manner, with Platycodi Radix extract having the greatest antibacterial effect. In addition, *P. gingivalis* that were treated with Platycodi Radix extract were significantly larger than those treated with Aurantii Fructus Immaturus or Paeoniae Radix extracts. Further analysis showed that platycodin D, which is one of the ingredients of Platycodi Radix, reduced bacterial growth. **Conclusions:** Platycodi Radix is the active component in Hainosan and may represent a useful agent for the treatment of *P. gingivalis*-induced gingivitis and periodontitis.

## 1. Introduction

*Porphyromonas gingivalis* (*P. gingivalis*) is a Gram-negative obligate anaerobic bacterium that is involved in the initiation and progression of oral inflammation diseases such as gingivitis and periodontitis [1]. This bacterium possesses many virulence factors, such as fimbriae and proteases, and its contribution to periodontal disease progression may be influenced by contact-dependent mechanisms [2]. Furthermore, the pathogenesis of gingivitis and periodontitis can also be affected by bacteria-derived metabolic products [3].

Hainosan (Painongsan) is a traditional Chinese medicine (TCM) that was first described in the *Jinguiyaolue* by Zhang Zhongjing in the Later Han Dynasty. This formulation is also used in traditional Japanese Kampo medicine to treat acute or chronic purulent diseases, including gingivitis and periodontitis [4]. Hainosan extract (HNS) is composed of three crude drugs: The dried immature fruit of *Citrus aurantium* (Aurantii Fructus Immaturus), the dried root of *Paeonia lactiflora* (Paeoniae Radix), and the dried root of *Platycodon grandiflorum* (Platycodon Radix) [5]. 

Antibiotic treatment is used as a standard therapy for bacterial infections, even in anaerobic bacterial infections [6]. *P. gingivalis* itself is often susceptible to β-lactam antibiotics, but recently, the presence of β-lactamase-producing *P. gingivalis* that degrades β-lactam antibiotics has become a problem [7]. Moreover, in the oral cavity, the β-lactam antibiotics are eventually degraded by β-lactamase-producing bacteria, such as *Prevotella intermedia* and *Moraxella catarrhalis* and may not reach *P. gingivalis*, which is considered to be the reason why the antibacterial treatment is not effective [8,9].

Thus, although periodontal disease is exacerbated by inflammation caused by *P. gingivalis*, no antibacterial agent against *P. gingivalis* is used directly in the treatment of periodontitis in Japan [10]. In the situation where there are few oral periodontitis treatments, HNS is an approved drug in Japan with the efficacy for periodontitis as an oral administration [11]. We already found that HNS may directly inhibit the bacterial growth and biofilm formation in vitro [12], although it remains unclear which crude drug components of HNS are effective on these anaerobic bacteria. The aim of the present study was to investigate the antibacterial effect of HNS and the extracts of its components against the anaerobic bacterium *P. gingivalis*.

## 2. Materials and Methods 

### 2.1. Crude Drugs and Chemicals

Aurantii Fructus Immaturus (lot # MC020B2-170D-003), Paeoniae Radix (MC041D1-170D-161), and Platycodi Radix (MC018C4-170D-001), standardized by the *Japanese Pharmacopoeia*, 17th Edition, were obtained from JPS Pharmaceuticals (Yokohama, Japan) [13]. To obtain powdered extracts of each product, Aurantii Fructus Immaturus (9.0 g), Paeoniae Radix (9.0 g), Platycodi Radix (4.5 g), or their mixture (HNS) were boiled in distilled water (450 mL) for 30 min and filtered, following which the decoction was lyophilized to yield dried powdered extracts (Aurantii Fructus Immaturus, 4.0 g; Paeoniae Radix, 4.4 g; Platycodi Radix, 5.7 g; and HNS, 8.0 g). These dried powdered extracts were then suspended in distilled water and stored at −20 °C until use. Platycodin D was purchased from Fujifilm Wako Pure Chemical (Osaka, Japan).

### 2.2. Bacterial Strains and Culture Conditions

*Porphyromonas gingivalis* JCM12257 (ATCC33277), JCM8525, and JCM19600 were purchased from RIKEN BioResource Research Center (Ibaraki, Japan) [14]. All three strains were grown at 37 °C under anaerobic conditions (AnaeroPack™-Anaero, Tokyo, Japan) in a Gifu anaerobic medium broth (Nissui, Tokyo, Japan) supplemented with 5 µg/mL hemin (Sigma-Aldrich, St. Louis, MO, USA) and 1 µg/mL menadione (Fujifilm Wako Pure Chemical, Osaka, Japan) (GAM medium). Ampicillin sodium (ABPC) (Fujifilm) was only used as a positive control at a final concentration of 50 µg/mL.

### 2.3. Growth-Inhibitory Analysis 

The bacteria were preincubated in CDC Anaerobe Blood Agar (Nihon BD, Tokyo, Japan) for 48 h. Approximately 1 × 10^6^ bacteria/mL were then incubated in 2 mL of GAM medium containing each of the extracts for 48 h, and the resulting colonies (numbers of colony-forming units (CFUs)) were counted to determine the growth-inhibitory activity of each product (HNS, crude drugs, and platycodin D or ABPC). Data from triplicate assays were averaged and plotted for each time point. All experimentation was carried out under anaerobic conditions.

### 2.4. Time-Kill Analysis 

A time-kill analysis was performed as described previously [15]. GAM medium containing one of the crude drug extracts, platycodin D or ABPC, was inoculated with bacterial suspension at a final concentration of 1 × 10^8^ CFU/mL in triplicate. Aliquots of undiluted and 10-fold serially diluted samples were then plated onto CDC Anaerobe Blood Agar at 0 and 30 min after inoculation and the plates were incubated at 37 °C for 48 h. The resulting colonies (number of CFUs) were then counted and data from triplicate assays were averaged and plotted for each time point. All experiments were carried out under anaerobic conditions.

### 2.5. Morphological Analysis

The bacterial morphology was investigated using transmission electron microscopy (TEM) [16]. First, bacteria (1 × 10^6^ CFU) that had been treated with HNS, one of the crude drug extracts, or ABPC were cultured in GAM medium for 48 h. Then, approximately one drop of the bacterial culture was applied to a Formvar/carbon-coated 300-mesh copper grid (Nisshin EM, Tokyo, Japan), the excess solution was removed, and 2% phosphotungstic acid (PTA) (Fujifilm) was added for negative staining. The samples were then observed under a transmission electron microscope (JEM1011J; JEOL, Tokyo, Japan) and digital images were taken with a MegaView Slow-scan camera (JEOL). We also measured the square area of bacterial shape by Image J [17]. The area was measured at 5 arbitrary points in bacteria treated with the crude drugs.

### 2.6. Statistical Analysis

An unpaired Student’s *t*-test was used to examine the differences between two groups. A Tukey’s multiple comparison test was used to assess differences among multiple groups (EZR version 1.36, http://www.jichi.ac.jp/saitama-sct/SaitamaHP.files/statmedEN.html). A *p* value of less than 0.05 was considered statistically significant.

## 3. Results

### 3.1. Growth-Inhibitory Analysis of HNS and the Crude Drug Extracts

**Figure 1:**
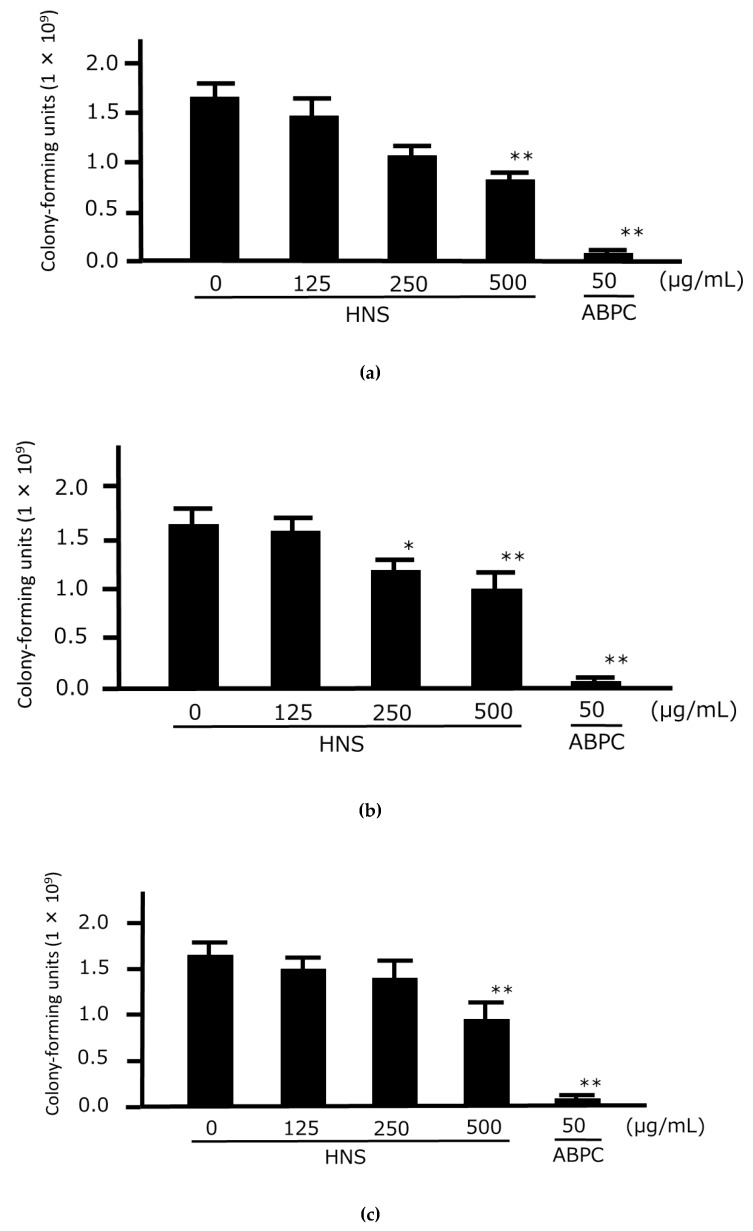
Growth-inhibitory effects of hainosan extract (HNS) against three *Porphyromonas gingivalis* isolates. *P. gingivalis* JCM12257 (**a**), JCM8525 (**b**), and JCM19600 (**c**) were treated with HNS for 48 h. An aliquot of each liquid culture was then inoculated onto a CDC anaerobic plate and cultured for 2 d in an anaerobic atmosphere, following which the number of colony-forming units was counted. Data points represent the numbers of viable cells in a culture medium or culture medium containing HNS and are means ± SD (n = 3). * *p* < 0.05, ** *p* < 0.01 vs. the untreated group (Tukey’s multiple comparison test).

**Figure 2:**
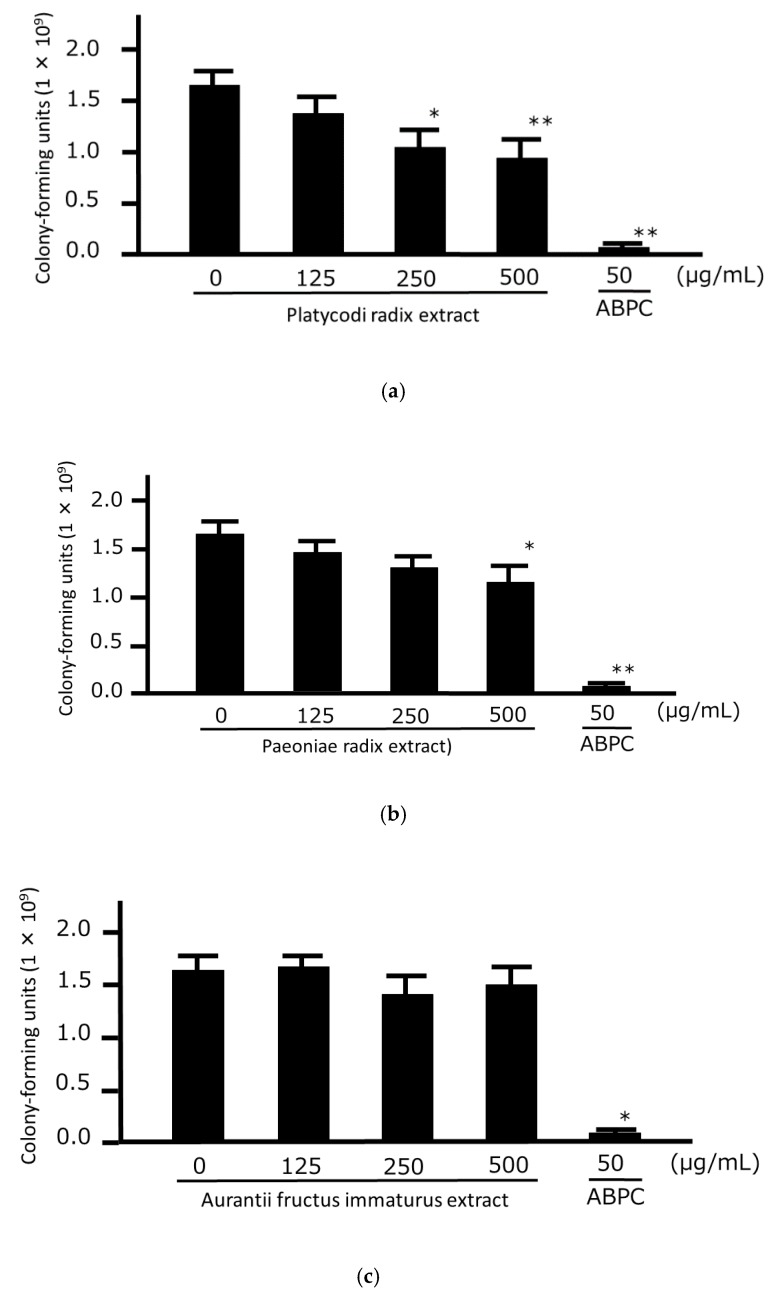
Growth-inhibitory effects of the three components of hainosan extract (HNS) against *Porphyromonas gingivalis*. *P. gingivalis* JCM12257 was treated with extracts of Platycodi Radix (**a**), Paeoniae Radix (**b**), or Aurantii Fructus Immaturus (**c**) for 48 h. An Aliquot of each liquid culture was then inoculated onto a CDC anaerobic plate and cultured for 2 d in an anaerobic atmosphere, following which the number of colony-forming units was counted. Data points represent the numbers of viable cells in a culture medium or culture medium containing HNS and represent means ± SD (n = 3). * *p* < 0.05, ** *p* < 0.01 vs. the untreated group (Tukey’s multiple comparison test).

First, we examined the concentration-dependent growth-inhibitory effects of HNS on the three strains of *P. gingivalis* to confirm the universality of its antibacterial effect against this bacterium. We found that the number of CFUs of all three strains of *P. gingivalis* were significantly lower when 250 and 500 µg/mL HNS were applied and we observed that HNS had direct antimicrobial effects against *P. gingivalis* (Figure 1). Next, we examined the direct growth-inhibitory effects of the extracts of three components of HNS against *P. gingivalis*. Significant antibacterial effects against the bacteria were observed at concentrations of 250 and 500 µg/mL in Platycodi Radix extract and a significant difference at a concentration of 500 µg/mL in Paeoniae Radix extract. No significant growth inhibitory effect of bacteria was observed for Aurantii Fructus Immaturus extract up to 500 µg/mL (Figure 2).

### 3.2. Time-Kill Analysis of the Crude Drug Extracts

**Figure 3:**
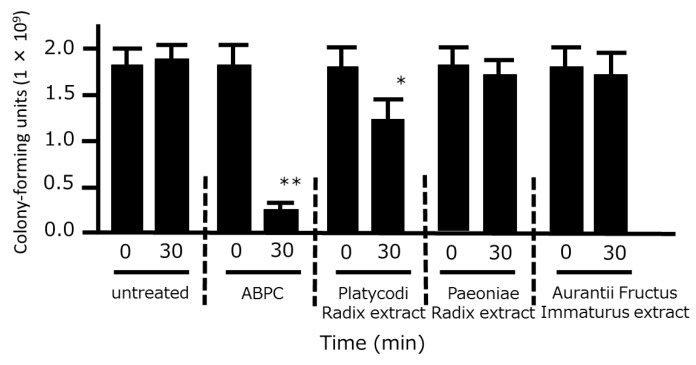
Time-kill analysis of the three extracts of HNS components against *Porphyromonas gingivalis*. *P. gingivalis* (1 × 10^6^ colony-forming units) was treated with ampicillin sodium (ABPC; 50 µg/mL), Platycodi Radix extract (500 µg/mL), Paeoniae Radix extract (500 µg/mL), and Aurantii Fructus Immaturus extract (500 µg/mL) for 30 min, and the number of colony-forming units was then counted. Data represent the numbers of viable cells in a culture medium. Data represent means ± SD (n = 3). * *p* < 0.05, **p < 0.01 vs. each 0 min group (Student’s *t*-test).

We also examined the time-kill effects of the three crude drug extracts on *P. gingivalis*. A significant bacterial reduction was observed after 30 min of treatment with Platycodi Radix extract, whereas no such effect was observed when bacteria were treated with Aurantii Fructus Immaturus or Paeoniae Radix extracts (Figure 3). 

### 3.3. Morphological Effects of the Crude Drug Extracts

**Figure 4:**
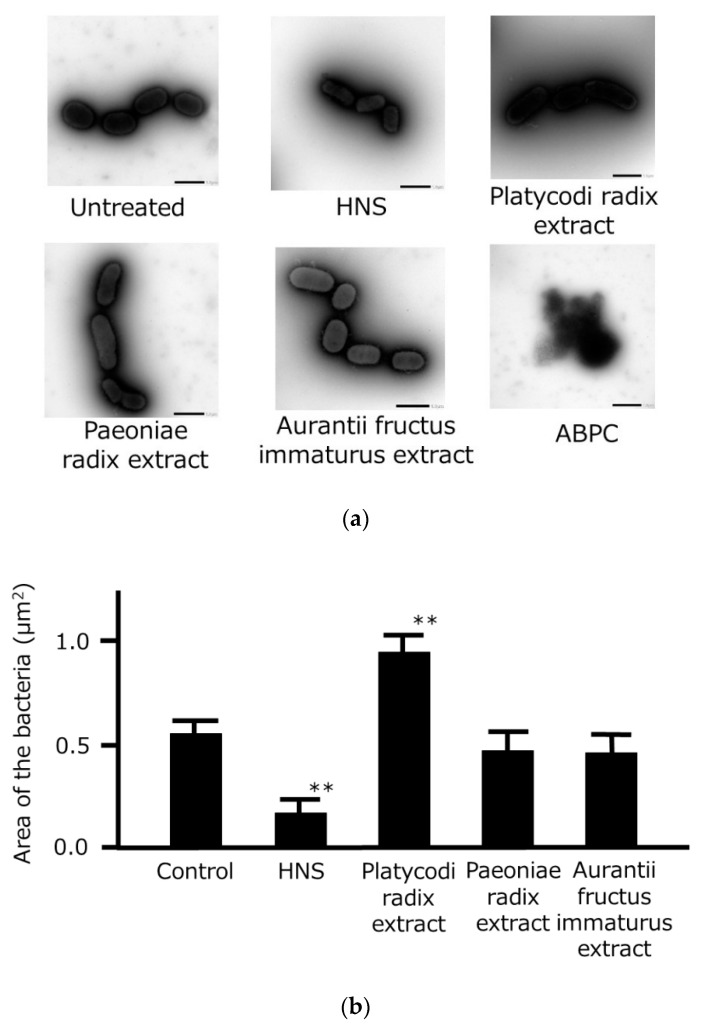
Morphological changes in *Porphyromonas gingivalis* following treatment with the three components of HNS. (**a**) Representative photographs of *P. gingivalis* following treatment with HNS, the extract of one of the three components of HNS (500 µg/mL), or ampicillin sodium (ABPC; 50 µg/mL) for 48 h, viewed under a transmission electron microscope with negative staining. Bars represent 1 µm. (**b**) Area of *P. gingivalis* treated with HNS, the extract of one of the three components of HNS (500 µg/mL), or ABPC (50 µg/mL) for 48 h. The area was measured at 5 arbitrary points in bacteria treated with the crude drugs. Data represent means ± SD (*n* = 5). ** *p* < 0.01 vs. the control group (Tukey’s multiple comparison test).

We examined the morphological effects of the three crude drug extracts on *P. gingivalis* using TEM (Figure 4). When treated with ABPC (positive control), the bacteria were completely destroyed and did not retain their shape. HNS treatment caused the bacteria to have a reduced area compared with untreated bacteria, whereas treatment with Platycodi Radix extract significantly increased the area of the bacteria compared with bacteria that had been treated with Aurantii Fructus Immaturus and Paeoniae Radix extracts.

### 3.4. Growth-Inhibitory and Time-Kill Analyses of Platycodin D

**Figure 5:**
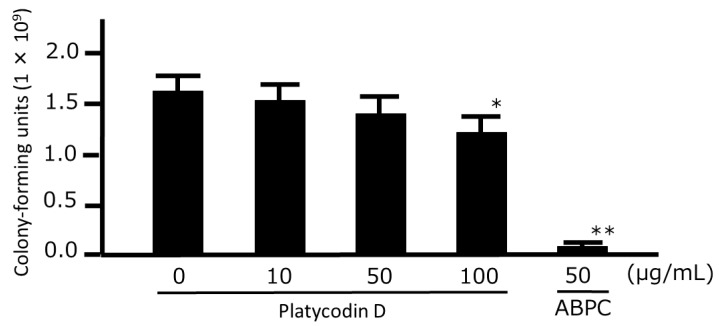
Growth-inhibitory effects of platycodin D against *Porphyromonas gingivalis*. *P. gingivalis* JCM12257 were treated with platycodin D for 48 h. An aliquot of the liquid culture was then inoculated onto a CDC anaerobic plate and cultured for 2 d in an anaerobic atmosphere, following which the number of colony-forming units was counted. Data points represent the numbers of viable cells in a culture medium or culture medium containing HNS and represent means ± SD (n = 3). * *p* < 0.05, ** *p* < 0.01 vs. the untreated group (Tukey’s multiple comparison test).

**Figure 6:**
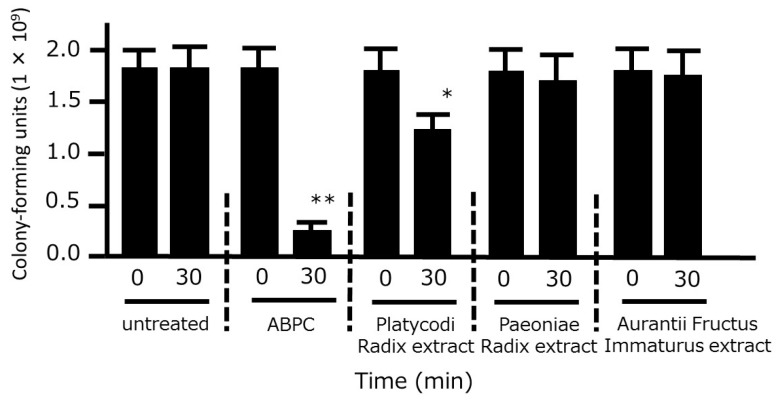
Time-kill analysis of platycodin D against *Porphyromonas gingivalis*. *P. gingivalis* JCM12257 (1 × 10^6^ colony-forming units) were treated with platycodin D (0, 10, 50, or 100 µg/mL), and ampicillin sodium (ABPC; 50 µg/mL) for 30 min. An aliquot of each liquid culture was then inoculated onto a CDC anaerobic plate and cultured for 2 d in an anaerobic atmosphere, following which the number of colony-forming units was counted. Data represent means ± SD (*n* = 3). * *p* < 0.05, ** *p* < 0.01 vs. each 0 min group (Student’s *t*-test).

To evaluate which ingredient of Platycodi Radix is effective against *P. gingivalis*, we examined the antibacterial effect of platycodin D, which was determined to be a marker component of Paeoniae Radix in the *Japanese Pharmacopoeia*, 17th Edition [13]. Platycodin D only had a significant growth-inhibitory effect on the bacteria at a concentration of 100 µg/mL, with concentrations of 50 µg/mL or less having no significant effects (Figure 5). Furthermore, a time-kill analysis similarly showed that only a concentration of 100 µg/mL caused a significant reduction in the number of bacteria after 30 min (Figure 6). 

## 4. Discussion

In this study, we examined the antibacterial effects of HNS and its crude drug components on *P. gingivalis*. We confirmed that HNS had a growth-inhibitory effect against all three strains of *P. gingivalis*. We also found that Platycodi Radix extract, which is a component of HNS, had growth-inhibitory and time-kill effects at a concentration of 250 µg/mL, while one of its signal components, platycodin D, exhibited these effects at only 100 µg/mL. 

Although HNS decreased the area of the bacteria, its component crude drugs, particularly Platycodi Radix, significantly increased the area, indicating that they cause bacterial swelling. Since HNS is a mixture of the three crude drugs, it is possible that the bacteria were partially broken as a result of excessive swelling and consequently contracted, which is supported by the fact that treatment with HNS significantly reduced the number of bacteria.

We next examined which component of Platycodi Radix had the antibacterial effect on the bacteria by treating the bacteria with commercially available platycodin D. We found that only a concentration of 100 µg/mL had a significant antibacterial effect, which is unlikely to be provided by Platycodi Radix since it only has a content of approximately 1% platycodin D [5,18]. Therefore, it seems likely that some other as yet unidentified components within Platycodi Radix are involved in its antibacterial effect. Since Platycodi Radix also contains some saponins, these should be the subject of further research [18]. 

Paeoniae Radix extract also showed some antibacterial effects against *P. gingivalis*, supporting the findings of a previous report [19]. *Porphyromonas gingivalis* has also been shown to experience a marked growth reduction in the presence of naringin, which is one of the constituents of Aurantii Fructus Immaturus, with no CFUs being observed after 24 h [20]. However, in the present study, Aurantii Fructus Immaturus extract did not inhibit the growth of *P. gingivalis*. These conflicting findings may have resulted from differences in the herbal medicines and bacteria but further study may be required in the future to clarify this. 

To date, very few studies have investigated the beneficial effects of Kampo medicines on periodontal disease. Shosaikoto (Xiaochaihutang) and Orento (Huangliansutang) suppress lipopolysaccharide (LPS)-induced prostaglandin E_2_ production by human gingival fibroblasts [21,22], while Juzentaihoto (Shiquandabutang) inhibits osteoclast differentiation in vitro and reduces alveolar bone loss in a rat periodontitis model [23]. Other study showed that Rokumigan (Liuweidihuangwan) promotes wound healing in a gingival fibroblast model, attenuates pro-inflammatory cytokine secretion by oral mucosal cells, and prevents biofilm formation by *Fusobacterium nucleatum* [24], while Daiokanzoto (Dahuanggancaotang) inhibits the growth and adherence properties of *P. gingivalis* [25]. Several medicinal plants are known to have antibacterial effects against *P. gingivalis*, such as *Robinia pseudoacacia*, *Phytolacca americana*, *Antrodia camphorata*, and *Syzygium aromaticumare* [26,27,28,29]. However, these investigations have only demonstrated growth-inhibitory effects as minimum inhibitory concentrations (MICs) and have not investigated bacterial morphological effects. By showing morphological changes in this study, we have more specifically demonstrated the anti-bacterial effect of HNS and its component of crude drugs. Therefore, in this study, the elucidation of part of the mode of action of HNS against periodontitis-associated bacteria will clinically advance our understanding of the antibacterial effects of crude drugs on bacteria.

## 5. Conclusions

Our findings indicate that Platycodi Radix may be a useful agent for the treatment of *P. gingivalis*-induced periodontitis.
